# Comparison of Microbial Communities Isolated from Feces of Asymptomatic *Salmonella*-Shedding and Non-*Salmonella* Shedding Dairy Cows

**DOI:** 10.3389/fmicb.2016.00691

**Published:** 2016-06-01

**Authors:** Bradd J. Haley, James Pettengill, Sasha Gorham, Andrea Ottesen, Jeffrey S. Karns, Jo Ann S. Van Kessel

**Affiliations:** ^1^Environmental Microbial and Food Safety Laboratory, Beltsville Agricultural Research Center, U.S. Department of Agriculture, Agricultural Research ServiceBeltsville, MD, USA; ^2^Division of Public Health Informatics and Analytics, Center for Food Safety and Nutrition, Office of Analytics and Outreach, U.S. Food and Drug AdministrationCollege Park, MD, USA; ^3^Division of Microbiology, Center for Food Safety and Nutrition, Office of Regulatory Science, U.S. Food and Drug AdministrationCollege Park, MD, USA

**Keywords:** *Salmonella enterica*, dairy cows, fecal microbiome, fecal microbiota, 16S rRNA gene sequencing, Salmonella Kentucky, Salmonella Cerro

## Abstract

In the United States *Salmonella enterica* subsp. *enterica* serotypes Kentucky and Cerro are frequently isolated from asymptomatic dairy cows. However, factors that contribute to colonization of the bovine gut by these two serotypes have not been identified. To investigate associations between *Salmonella* status and bacterial diversity, as well as the diversity of the microbial community in the dairy cow hindgut, the bacterial and archaeal communities of fecal samples from cows on a single dairy farm were determined by high-throughput sequencing of 16S rRNA gene amplicons. Fecal grab samples were collected from two *Salmonella*-positive cows and two *Salmonella*-negative cows on five sampling dates (*n* = 20 cows), and 16S rRNA gene amplicons from these samples were sequenced on the Illumina MiSeq platform. A high level of alpha (within) and beta diversity (between) samples demonstrated that microbial profiles of dairy cow hindguts are quite diverse. To determine whether *Salmonella* presence, sampling year, or sampling date explained a significant amount of the variation in microbial diversity, we performed constrained ordination analyses (distance based RDA) on the unifrac distance matrix produced with QIIME. Results indicated that there was not a significant difference in the microbial diversity associated with *Salmonella* presence (*P* > 0.05), but there were significant differences between sampling dates and years (Pseudo-*F* = 2.157 to 4.385, *P* < 0.05). Based on these data, it appears that commensal *Salmonella* infections with serotypes Cerro and Kentucky in dairy cows have little or no association with changes in the abundance of major bacterial groups in the hindgut. Rather, our results indicated that temporal dynamics and other undescribed parameters associated with them were the most influential drivers of the differences in microbial diversity and community structure in the dairy cow hindgut.

## Introduction

The microbial community of the gastrointestinal system of dairy cows remains an understudied environment. Due to its potential impact on animal health, nutrient uptake, productivity, potential to serve as a reservoir of human and animal pathogens, as well as overall animal health, there is a need to better understand bovine gut microbial communities. Both human and animal pathogens can be found in this environment in low to high concentrations. This remains a public health issue in both developed and developing nations where dairy cows represent a significant food source providing both milk and meat. Further, carriage by animals can result in continuous dissemination of human and animal pathogens to the environment (Nightingale et al., 2004; Travier et al., 2013), other animals (via direct contact, environmental or wildlife carriage) (Wells et al., 2001; Nightingale et al., 2004; Böhm et al., 2009; Spencer et al., 2015), or food products at slaughter (Omisakin et al., 2003). Therefore, it is important to better understand the potentially supportive or inhibitive dynamic in the microbial communities in which these pathogens are found.

*Salmonella enterica* subsp. *enterica* serotypes Cerro and Kentucky are frequently isolated from feces of asymptomatic dairy cows in the United States. These serotypes are infrequent pathogens of humans, however, recent human salmonellosis cases attributed to these two serotypes have been reported (Bonalli et al., 2012; Centers for Disease Control Prevention, 2013). While other *Salmonella* serotypes such as Dublin, Newport, and Typhimurium can cause severe disease in cows (Poppe et al., 1998; Tsolis et al., 1999; Cobbold et al., 2006; Cummings et al., 2009a,b), which may be subsequently treated with antibiotic therapy, *S*. Cerro and *S*. Kentucky infections generally go unnoticed. Infected cows may potentially enter the food supply resulting in an increased risk to humans via consumption of contaminated products.

Previous work from a single commercial dairy farm in Pennsylvania demonstrated that both *S*. Cerro and *S*. Kentucky were repeatedly isolated from cows over a 6-year period (Van Kessel et al., 2012). During this time the prevalence of each serotype in fecal grab samples ranged between 0 and 90% indicating a wide variability of the within-herd prevalence of these serotypes (Van Kessel et al., 2012). These serotypes have also been detected frequently in cross sectional surveys of other dairy herds, but their prevalence was low (Huston et al., 2002; USDA, 2011; Loneragan et al., 2012). Regardless of prevalence among individuals within a herd, it is becoming more apparent that *S*. Cerro and *S*. Kentucky are successful at establishing residency within the bovine gut.

Recent advances in microbial community analysis have demonstrated that the microbiome plays a role in the modulation of colonization by an infectious bacterium (Chang et al., 2008; Koch and Schmid-Hempell, 2011; Reeves et al., 2011; Buffie and Pamer, 2013; Britton and Young, 2014). The host microbial community may prevent colonization by a pathogen or enhance the susceptibility of the host to colonization and infection, and colonization may ultimately alter the intestinal microbial community profile of the host (Hopkins and Macfarlane, 2002; Aebischer et al., 2006; Stecher et al., 2007; Nelson et al., 2012; Britton and Young, 2014). To date, several microbial community analyses of the bovine gastrointestinal tract using high-throughput sequencing have been conducted (Dowd et al., 2008; Callaway et al., 2010; Durso et al., 2010; Shanks et al., 2011; Rice et al., 2012; Rudi et al., 2012; Kim et al., 2014; Mao et al., 2015; Myer et al., 2015; Kim and Wells, 2016). However, differences in management practices between farms, environmental pressures, and even bovine genetics suggest a greater diversity exists than what has been reported. Further, the relationship between commensal *Salmonella* serotypes and the bovine hindgut microbial community has not been well-described. The objectives of this study were to further expand our understanding of the microbial community of the dairy cow hindgut and potentially identify the microbial interactions that influence presence/absence of commensal *Salmonella* serotypes. We profiled the prokaryotic community, using 16S rRNA gene sequencing, from feces of 20 lactating dairy cows from a typical commercial dairy operation in south-central Pennsylvania, USA. Of these 20 cows, 10 were shedding *S*. *enterica* serotypes Cerro or Kentucky and 10 were not. Furthermore we provided a valuable baseline description of the microbial community structure of the hindgut of 20 dairy cows.

## Materials and methods

### Sample collection and processing

Fecal grab samples were collected from lactating dairy cows on a commercial dairy farm in south-central Pennsylvania, USA. Animal sampling procedures were approved by The Pennsylvania State University Institutional Animal Care and Use Committee (Protocol #44324). Samples were transported to the laboratory in Beltsville, MD in sterile 50 ml conical tubes on ice (*ca*. 3 h), stored over night at 4°C, and processed the following day for *S. enterica* as previously described (Van Kessel et al., 2012). To determine whether cows were shedding *S. enterica*, traditional microbiological methods were conducted. Briefly, fecal samples were mixed with buffered peptone water (BPW) and homogenized. An aliquot of the fecal/BPW mixture was preserved for further analysis at –80°C. Samples were enriched in tetrathionate broth (BD Diagnostics, Sparks, MD) and struck onto XLT4 agar (XLT4 agar base with XLT4 supplement; BD Diagnostics) for isolation and incubated at 37°C overnight. Presumptive *S. enterica* colonies were transferred onto XLT4 plates, Brilliant Green, and L-Agar (Lennox Broth Base with 1.5% agar; Gibco Laboratories, Long Island, NY) and incubated at 37°C for 24 h. Colonies exhibiting the *Salmonella* phenotype were confirmed using the *invA*-PCR method of Rahn et al. (1992) as described by Malorny et al. (2003) and serogroups were molecularly determined using the methods of Herrera-León et al. (2007) with modifications by Karns et al. (2015). Enriched biomass was also tested for the presence of *Salmonella* using the real time PCR method described in Van Kessel et al. (2011).

Samples were selected for the current study to represent 10 cows that were shedding *Salmonella* and 10 cows that were identified as *Salmonella* negative (Table 1). Cows were selected as *Salmonella*-positive if *S. enterica* was detected in their feces by traditional culture analysis and real-time PCR detection of the *invA* gene. Cows negative for *Salmonella* for both assays were chosen as *Salmonella*-negative cows. Matching samples (*Salmonella*-negative, *Salmonella*-positive) were selected from samples collected in January 2006, March 2006, December 2008, December 2009, and March 2012. Sampling dates were selected from archived samples to capture the appropriate number of *Salmonella*-shedding and non-shedding cows to conduct the analysis. Total DNA was extracted from preserved fecal grab samples using a QIAamp Fast DNA Stool Mini Kit (Qiagen, Hilden, Germany) and DNA was evaluated for quality using a Nanodrop 2000 (Thermo Fisher Scientific, Waltham, MA) and a Qubit 2.0 (Thermo Fisher Scientific, Waltham, MA).

**Table 1:** **Sample ID, collection date, and *Salmonella* status of samples collected from dairy cows**.

**Sample ID**	**Date**	***Salmonella* status**	**MG-RAST accession**
CFSANAZA0001	Jan-06	NEG	4681773.3
CFSANAZA0002	Jan-06	POS	4681767.3
CFSANAZA0003	Jan-06	POS	4681782.3
CFSANAZA0004	Jan-06	NEG	4681780.3
CFSANAZA0005	Mar-06	NEG	4681779.3
CFSANAZA0006	Mar-06	POS	4681764.3
CFSANAZA0007	Mar-06	NEG	4681765.3
CFSANAZA0008	Mar-06	POS	4681770.3
CFSANAZA0009	Dec-08	NEG	4681778.3
CFSANAZA0010	Dec-08	NEG	4681772.3
CFSANAZA0011	Dec-08	POS	4681771.3
CFSANAZA0012	Dec-08	POS	4681781.3
CFSANAZA0013	Dec-09	NEG	4681769.3
CFSANAZA0014	Dec-09	NEG	4681766.3
CFSANAZA0015	Dec-09	POS	4681777.3
CFSANAZA0016	Dec-09	POS	4681776.3
CFSANAZA0017	Mar-12	NEG	4681768.3
CFSANAZA0018	Mar-12	NEG	4681774.3
CFSANAZA0019	Mar-12	POS	4681775.3
CFSANAZA0020	Mar-12	POS	4681763.3

### 16S rRNA gene tailed amplicon sequencing

16S rRNA gene amplicon sequencing was performed according to the Illumina “*Overview of tailed amplicon sequencing approach with MiSeq*” protocol (http://www.Illumina.com). This two-step PCR utilizes sequence-specific primers and the Nextera DNA Index Kit (Illumina, San Diego, CA). Sequence-specific primers (IDT Inc., Coralville, Iowa) were designed according to low diversity amplicon specifications. Adapter overhang sequences TCGTCGGCAGCGTCAGATGTGTATAAGAGACAG and GTCTCGTGGGCTCGGAGATGTGTATAAGAGACAG were added to the 5′ end of the forward and reverse primers, respectively. These 5′-primer regions are complementary to sequences within the Nextera DNA indices thus permitting the addition of a unique sample index and P5/P7 adapters to make the template compatible for hybridization to the flow cell.

Sequence-specific primers targeting the V4 hypervariable region of the 16S rRNA gene used for the first round of PCR were as follows: F515—5′ GTGCCAGCMGCCGCGGTAA 3′ and R806—5′ GGACTACHVGGGTWTCTAAT 3′ of Caporaso et al. (2012). Emerald Green GT PCR Master Mix (Takara Bio Inc. Otsu, Shiga, Japan) was used to generate amplicons. Negative controls with no template were run alongside sample DNA. Thermocycler settings used for PCR were as follows: 95°C 3 min, 94°C 1 min, 56°C 1 min, 72°C 1 min, cycle 29 times, 72°C 5 min, 4°C forever. PCR samples were run on a 2% agarose E-gel® (Invitrogen, Carlsbad, CA) with a 100 bp ladder (Invitrogen, Carlsbad, CA). Clean PCR product was obtained using AMPure XT® Beads (Beckman Coulter Inc. Brea, CA) to remove fragments smaller than 100 bases.

Two microliters of product from the first round of PCR was used as template for the second round of PCR. One microliter of each index N50X and N70X was added to the PCR reaction. Each sample had a different combination of N50X and N70X indices and there were no repeats. PCR was performed using the same thermocycler settings from the first round of PCR. Product obtained from the second round of PCR was cleaned using AMPure XT® beads and sample DNA concentration was determined using the Qubit® High-Sensitivity Assay (Life Technologies, Grand Island, NY).

Samples were then diluted to 2 nM DNA with EB buffer (Qiagen, Hilden, Germany) and pooled. Six hundred microliters of the combined sample at a concentration of 5 pM was loaded onto a MiSeq V2 500 cycle cartridge (Illumina, San Diego, CA). Twenty samples were sequenced on the MiSeq V2 platform.

### Quality filtering of 16S rRNA gene sequencing reads

For each sample, paired end reads were Merged using the program FLASH (Magoč and Salzberg, 2011) with the default settings (e.g., a minimum of 10 bp overlap) except for the maximum overlap expected which was increased from 65 to 500 bp to better accommodate our 251 bp read length. We then combined the file with merged reads and only the read 1 file; we chose to use just the “not combined” read 1 file because read 1 tends to be of higher quality than read 2 and by not using both “not combined” files we minimize inaccurately estimating abundance. We used the program SolexaQA to quality filter the reads with a probability cutoff value of a basecall being an error of 0.05. To further quality filter the reads, we also filtered the sequences for primers using Trimmomatic v0.33 (Schmieder et al., 2010) and removed the following: any reads < 100 bp; reads assigned to PhiX using custom scripts and BLAST; chimeric sequences using QIIME v1.8 (Caporaso et al., 2010a) with usearch (Edgar et al., 2011); and sequences assigned to chloroplast or mitochondrial genomes using mothur v1.33 (Schloss et al., 2009). The above filtering steps reduced the average number of reads per sample by ~13% from 138,295 to 120,080.

### Taxonomic assignment and detecting differences in microbial diversity

We used the program QIIME v1.8 and the pick_open_reference_otus.py method to estimate microbial diversity. This included using the Greengenes taxonomy (v13_8_99; McDonald et al., 2012) as the reference database, PyNast (Caporaso et al., 2010b) to align sequences and FastTree (Price et al., 2010) to construct a phylogenetic tree that was then used to estimate UniFrac (Lozupone and Knight, 2005) distances. With these distances we then performed tests to detect differences in microbial diversity among the samples and different treatments (e.g., *Salmonella* status, sampling year, and sampling date).

We used three different approaches to investigate the differences in microbial diversity among the hindgut samples. The first approach was to use the weighted (i.e., incorporates estimates of abundance) UniFrac distance matrix with the ordination technique, PCoA, implemented in QIIME to determine whether samples associated with the same group (year, date, *Salmonella_*status) clustered close to one another in multivariate space. The second approach was to use the constrained ordination technique distance-based redundancy analysis (db-RDA) to explicitly test whether the different factors (*Salmonella* status, sampling year, sampling date) explained a significant fraction of the variation within the distance matrix, which was implemented using the R package vegan called by QIIME with the compare_categories.py script. The third approach was to investigate if specific taxa significantly differed in abundance between the factors, which again was performed using QIIME (the group_signficance.py script). We chose the Kruskal–Wallis test as it is a non-parametric implementation of analysis of variance under which the assumptions of normality are relaxed (e.g., homoscedasticity). We defined the core microbiome as those genus-level taxa identified at least once in all samples for each group [all 20 samples, or all samples within a sample collection date (4 per date for 5 dates)]. Sequence data have been made publically available in MG-RAST under accession numbers 4681763.3 to 4681782.3.

## Results

### Trends in microbial community composition

**Figure 1:**
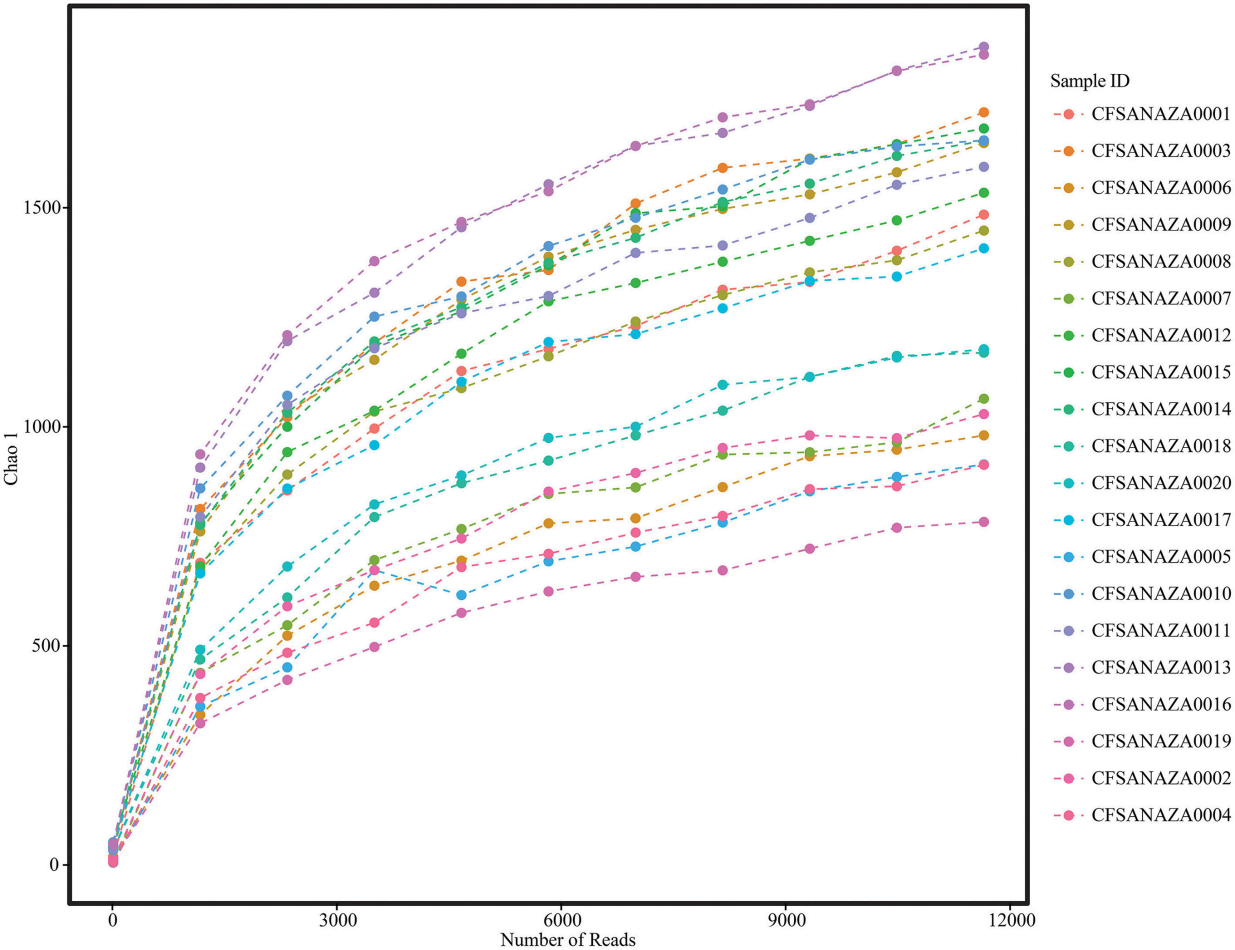
**Rarefaction plot showing the increase in alpha diversity (as measured by Chao 1) with increasing sequencing depth per sample**.

After filtering for quality, length, and removal of chimera sequences as well as those similar to the PhiX control, chloroplast and mitochondrial nucleic acids, analyzed sequences ranged between 2.79 × 10^4^ and 3.04 × 10^5^ (mean = 1.2 × 10^5^, median = 1.06 × 10^5^; Table 2). Rarefaction plots and Good's coverage statistic (both performed in QIIME) indicated that additional sequencing was unlikely to result in an increase in the diversity we observed (Figure 1; Table 2).

**Table 2:** **Sequencing statistics for dairy cow fecal grab samples**.

**CFSAN_ID**	**Number of raw reads**	**Number flashed**	**% Flashed**	**Reads >100bp**	**Hits To PhiX**	**Seqs remaining after Tag cleaned 100 bp filter**	**% remaining**	**Number of chimeric sequences**	**% chimeric**	**Number of chloroplasts and mitochondria**	**Final number of sequences for QIIME**	**% of original to QIIME**	**Number sequences passing QIIME prefilter**	**Good's coverage**
CFSANAZA0001	199,111	195,739	98.31	188,297	1	188,064	94.45	2414	1.21	33	185,617	93.22	185,556	0.9953
CFSANAZA0002	89,389	79,785	89.26	77,086	7	76,028	85.05	895	1	9	75,124	84.04	75,080	0.9913
CFSANAZA0003	143,367	130,852	91.27	118,607	7	116,635	81.35	1302	0.91	27	115,306	80.43	115,203	0.9888
CFSANAZA0004	45,922	34,091	74.24	28,347	19	28,162	61.33	165	0.36	0	27,997	60.97	27,942	0.986
CFSANAZA0005	194,780	192,134	98.64	188,170	2	188,031	96.54	1799	0.92	27	186,205	95.6	186,195	0.9968
CFSANAZA0006	104,566	96,576	92.36	90,742	9	88,752	84.88	982	0.94	15	87,755	83.92	87,688	0.9918
CFSANAZA0007	111,640	104,437	93.55	96,881	4	95,218	85.29	827	0.74	10	94,381	84.54	94,273	0.9916
CFSANAZA0008	69,867	48,048	68.77	44,098	81	43,880	62.81	580	0.83	17	43,283	61.95	43,212	0.9667
CFSANAZA0009	202,357	199,869	98.77	193,851	5	193,605	95.67	2081	1.03	23	191,501	94.64	191,401	0.9951
CFSANAZA0010	135,166	125,542	92.88	117,715	13	116,665	86.31	647	0.48	2	116,016	85.83	115,948	0.9882
CFSANAZA0011	129,717	127,109	97.99	123,581	1	123,412	95.14	616	0.47	9	122,787	94.66	122,730	0.9927
CFSANAZA0012	85,438	78,524	91.91	74,551	11	73,843	86.43	577	0.68	11	73,255	85.74	73,207	0.9843
CFSANAZA0013	128,555	116,658	90.75	104,613	2	103,048	80.16	985	0.77	13	102,050	79.38	101,992	0.9848
CFSANAZA0014	50,124	32,405	64.65	32,506	73	32,301	64.44	182	0.36	1	32,118	64.08	32,065	0.9537
CFSANAZA0015	321,863	316,977	98.48	307,669	5	307,265	95.46	3074	0.96	171	304,020	94.46	303,985	0.997
CFSANAZA0016	131,742	119,245	90.51	106,663	1	104,827	79.57	584	0.44	29	104,214	79.1	104,166	0.9871
CFSANAZA0017	161,232	148,316	91.99	145,908	14	144,880	89.86	1279	0.79	31	143,570	89.05	143,382	0.9928
CFSANAZA0018	98,499	52,526	53.33	67,643	29	67,165	68.19	467	0.47	14	66,684	67.7	66,600	0.9865
CFSANAZA0019	234,716	232,862	99.21	226,649	5	226,423	96.47	2820	1.2	12	223,591	95.26	223,565	0.9975
CFSANAZA0020	127,839	120,581	94.32	109,239	4	108,324	84.73	834	0.65	5	107,485	84.08	107,419	0.9922
Averages	138,294	127,613	88.56	122,140	15	121,326	83.71	1156	0.76	23	120,147	82.93	120,080	0.988

*Table shows number of reads before and after filtering for quality, length, chimeric sequences, chloroplasts, and mitochondria. Good's coverage is reported in the far right column*.

**Figure 2:**
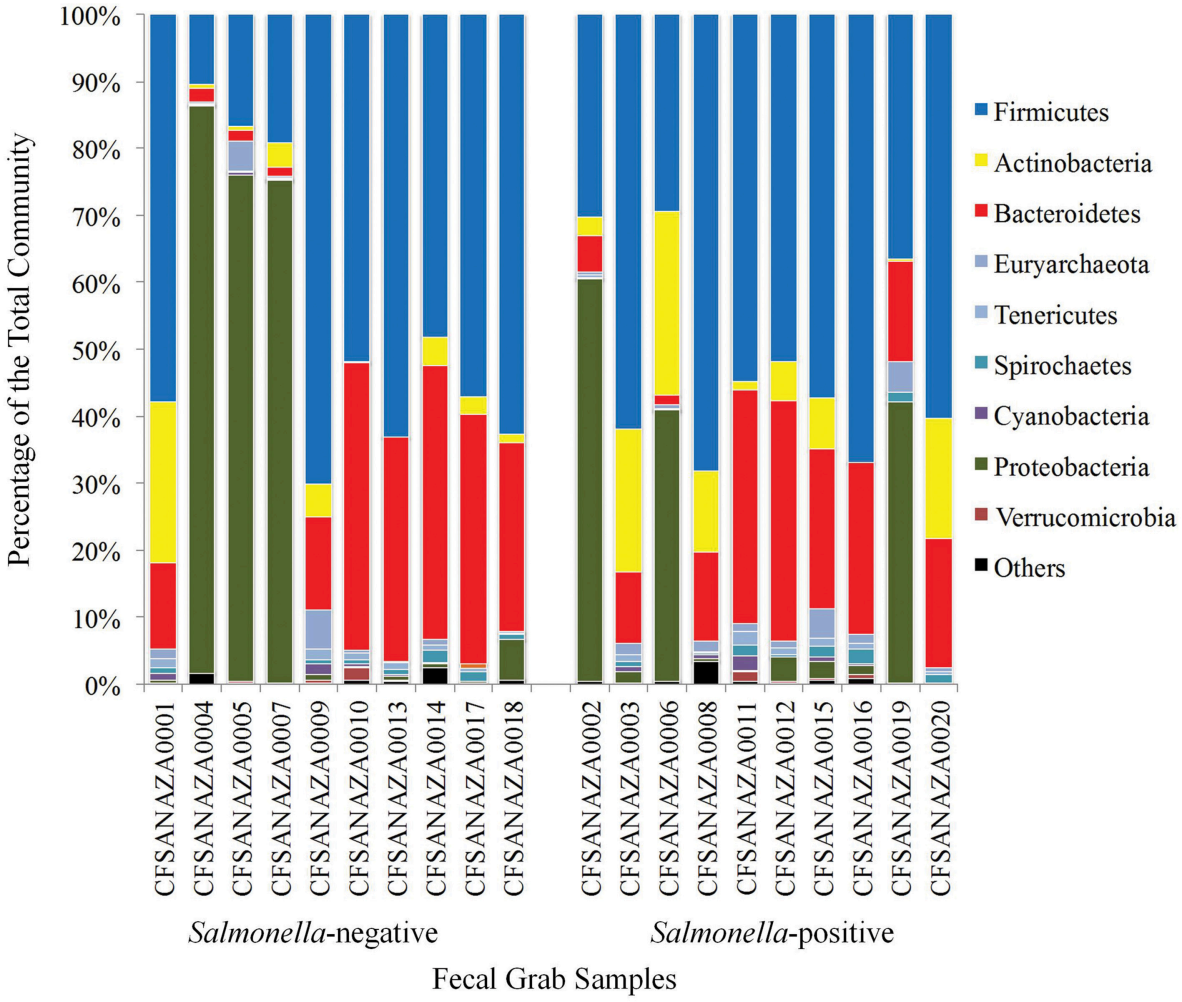
**Community profiles of the core phyla of the hindguts of individual cows**. The X-axis shows individual cows and the Y-axis shows percentage of hits to a particular phylum. In this figure *Salmonella*-status is based on results of traditional culture-based methods.

Taxa composition of fecal samples collected from different cows on the same dairy farm over time varied across the 6 years and within the same sampling period (Figure 2). For all but six samples, Firmicutes was the most abundant phylum detected (Figure 2 and Supplementary Table 1). For the six samples in which Firmicutes were not the most abundandant taxa, Proteobacteria taxa were most abundant, with Firmicutes as the second most abundant group. Bacteroidetes and Actinobacteria were also frequently detected in all samples. Previous studies have shown that taxa within Proteobacteria are well-represented in the bovine hindgut, but at concentrations typically < 5% of the community (Durso et al., 2010, 2012), however, others have shown the concentration of these organisms to be higher in various sections of the bovine gastrointestinal tract (Mao et al., 2015). In this study Proteobacteria was the most abundant phylum in six samples, comprising from 40 to 84% of all annotated taxa. Since this is much higher than what has been previously observed in the bovine hindgut (Dowd et al., 2008; Patton et al., 2009; Durso et al., 2010, 2012; Mao et al., 2015) we labeled these samples “atypical communities” and statistical analyses were conducted both including and excluding these six samples.

Within the Firmicutes, Proteobacteria, and Bacteroidetes phyla, Clostridia, Gammaproteobacteria, and Bacteroidia were the dominant classes, respectively. However, within each of these groups taxonomic trends differed noticeably between samples (Supplementary Table 1). The family with the highest median abundance within the Gammaproteobacteria class was the *Succinivibrionaceae* of the Aeromonadales order (median abundance = 0.09%, range = 0.01–1.3%). The *Moraxellaceae* had the highest mean abundance within the Gammaproteobacteria group, but this was mostly reflective of the high abundance of this family in the six atypical communities (range = 40–84%), and not reflective of their abundance within the other 14 samples (range = 0.008–0.20%). Within this family, *Psychrobacter* spp. were the most abundant genera and this group was driving the overabundance of Proteobacteria. It should be noted that *Psychrobacter* spp. were detected in all samples, while *Acinetobacter* spp. were detected in only seven samples (range = 0–0.008%). Twelve families were identified within the Clostridia, seven (*Ruminococcaceae, Lachnospiraceae, Clostridiaceae, Mogibacteriaceae, Peptostreptococcaceae, Veillonellaceae*, and the *Christensenellaceae*) of which were found in all samples (Supplementary Table 1). Of these, the *Ruminococcaceae* had the highest median detection frequency (median = 23%, range = 3–40%) but were not the most frequently detected member of the Clostridia class in each sample. Within the Bacteroidia class (Bacteroidetes phylum), 12 families were identified within the samples. Of these, eight were detected in all samples. These were *Bacteroidaceae, Paraprevotellaceae, Rikenellaceae, Porphyromonadaceae, Prevotellaceae*, and the uncultured families S24-7, RF16, and p-2534-18B5. In the *Prevotellaceae* family the only genus detected was *Prevotella*, often detected in beef cattle in high abundance (Durso et al., 2010, 2012). This genus was detected in all samples, but at a median percentage of only 0.08% of sequences. Other studies have similarly noticed a lower abundance of *Prevotella* spp. in dairy cows than in beef cows (Dowd et al., 2008; Durso et al., 2010).

*Enterobacteriaceae* were detected in all but one sample and at very low levels of abundance (median = 0.02%, range = 0–2.9%). Within this family unclassified members were the most abundant taxa. *Salmonella* spp. were detected in three samples that were *Salmonella* culture-negative, three samples that were *Salmonella* culture positive, but were not detected in seven samples that were *Salmonella* culture-positive.

### Core microbial communities

In an attempt to describe core members of the hindgut community we identified the taxa observed in all cows. Out of a total of 21 detected phyla, only nine were identified in all cows. These were Verrucomicrobia, Tenericutes, Spirochaetes, Proteobacteria, Firmicutes, Euryarchaeota, Cyanobacteria, Bacteroidetes, Actinobacteria (Supplementary Table 1). There were 11 classes of a total of 41 detected in all animals. One hundred thirty three different families were identified in all 20 samples. Of these, 30 were determined to be core families of the study farm hindgut microbiome. Of these, five could not be classified past the Order level and three were grouped into not-well-described families such as *Bacteroidales* S24-7, p-2534-18B5, and RF16 families.

**Figure 3:**
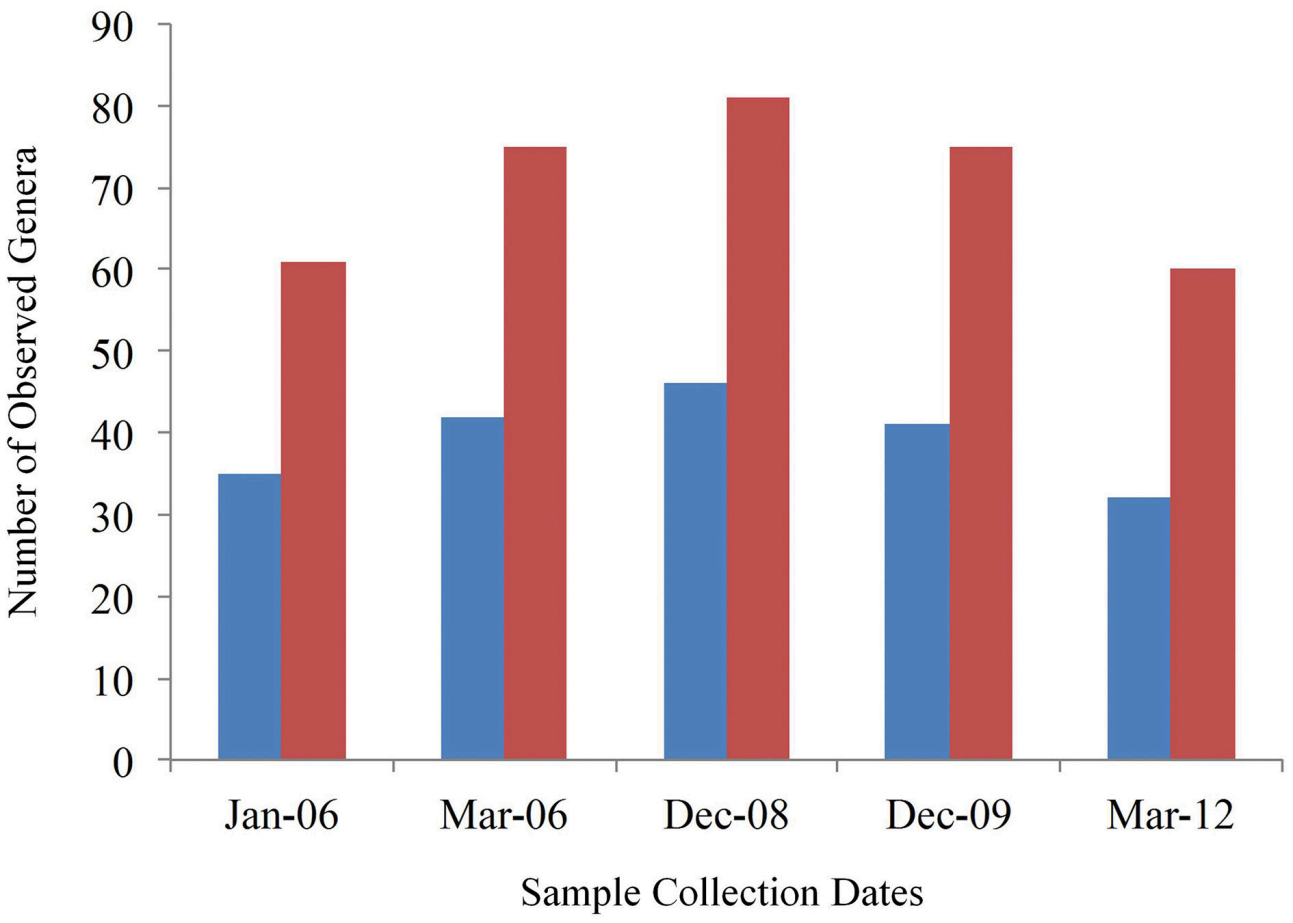
**Number of core genera per sampling date. Red bars are total genera categories and blue bars are number of classified genera**. The Y-axis shows the number of genera detected and the X-axis shows the sampling date. Four cows were sampled at each date.

Of the 253 identified genera, 46 were identified in all samples. However, only 26 could be classified in known genera while 20 grouped in undefined genera within known families. The number of core genera per sampling period varied as well, with 61 categories (including those that could only be typed to the family level) in January 2006, 75 in March 2006, 81 in December 2008, 75 in December 2009, and 60 in March 2012. When only sequences that could be assigned to a known genus were counted the core genera at each time point was 35 (January 2006), 42 (March 2006), 46 (December 2008), 41 (December 2009), and 32 (March 2012; Figure 3).

### Comparison of *Salmonella*-status and dates of isolation

**Figure 4:**
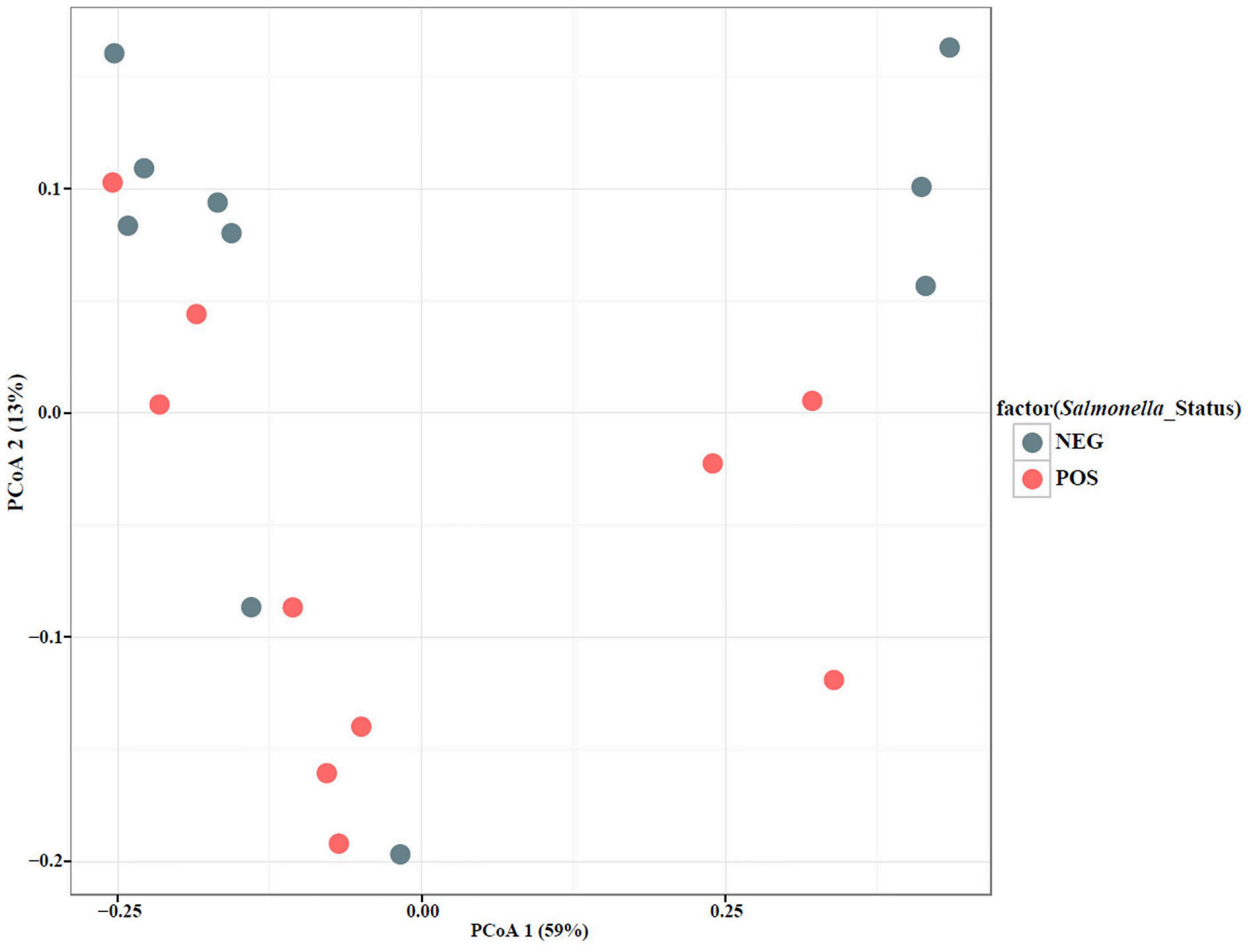
**Principal Coordinate Analysis (PCoA) plot based on UniFrac distances with samples color-coded by *Salmonella*-negative (Blue) and -positive (Red)**.

**Figure 5:**
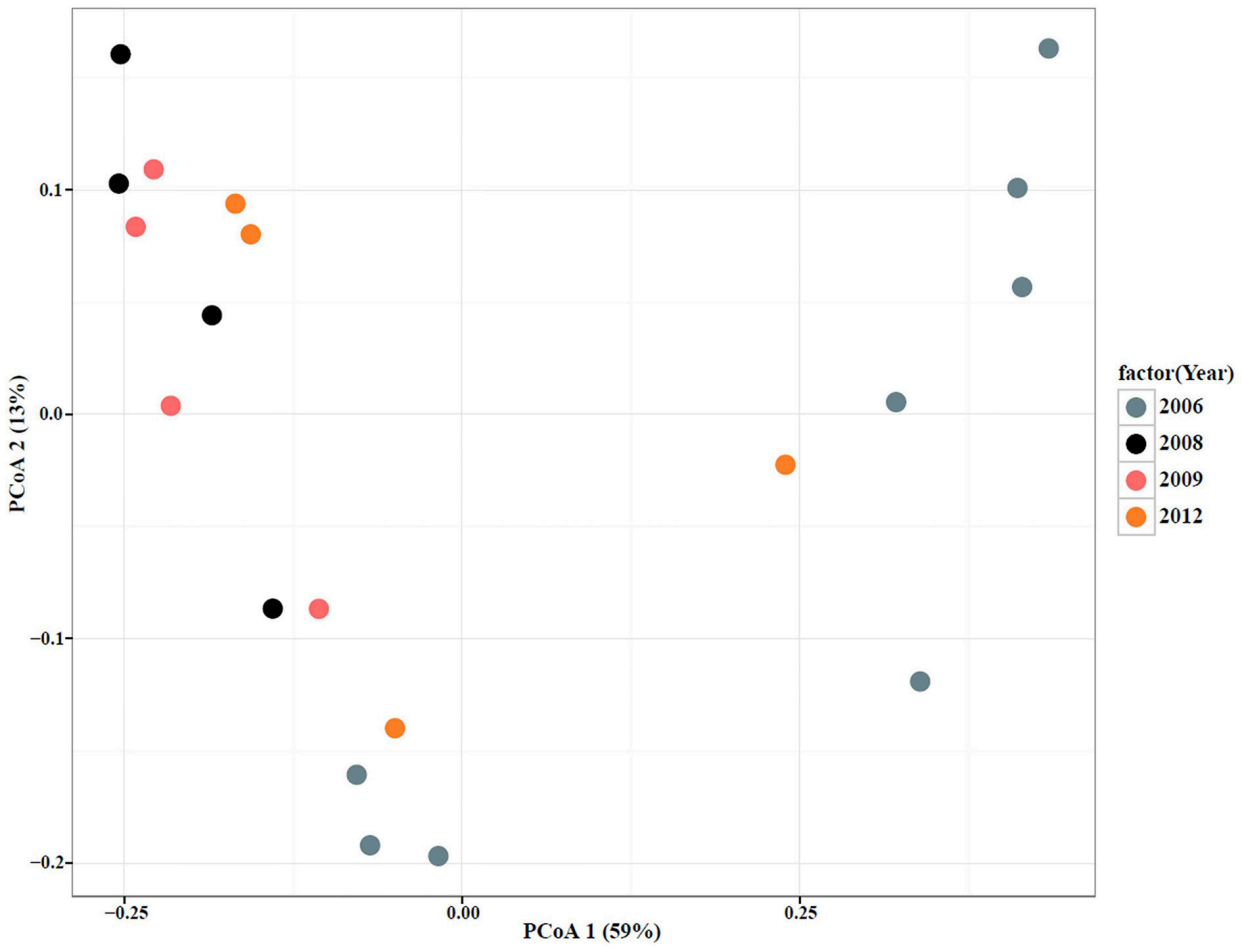
**Principal Coordinate Analysis (PCoA) plot based on UniFrac distances with samples color-coded by sampling year (Blue, 2006; Black, 2008; Pink, 2009; Orange, 2012)**.

To qualitatively investigate the differences in microbial diversity among samples within groups (year, date, and *Salmonella* status) a principal coordinate analysis (PCoA) was conducted. The PCoA results showed that a large percentage (59%) of the variation in the data could be explained by the first axis (Figures 4, 5). Generally, there appear to be two groups that are differentiated along that axis. However, when looking at the distribution of points in multivariate space with respect to group membership it is difficult to determine the cause of the large amount of variation. For example, there is little differentiation among samples that tested positive for *Salmonella* and those that did not. There also does not appear to be a pattern of differentiation among samples based on sample date; different years are found in close proximity to one another. PCoA axis 2 explained only 13% of the variation and there are no apparent differences among samples that correspond to the groups (year, date, *Salmonella*-status; Figures 4, 5). One noticeable trend is that samples with atypical communities were found in close proximity to each other in the PCoA analysis (Figures 4, 5).

There were no taxa identified in the *Salmonella*-positive samples that were not present in the *Salmonella*-negative samples, and vice versa, suggesting that presence of *Salmonella* was not associated with loss or absence of a specific group, or that our methods did not provide the resolution to discern this. Further, although based on a Kruskal–Wallis test the abundance of several OTUs were significantly different between *Salmonella* culture-positive samples and *Salmonella* culture-negative samples (α = 0.05), those results were not significant based on either the FDR or Bonferroni methods that account for multiple comparisons (Supplementary Table 2). This was true when the samples with atypical communities (i.e., those with extremely high abundance of Proteobacteria) were removed (Supplementary Table 3) and when *Salmonella* culture-negative but *Salmonella* 16S rRNA-positive were grouped together with *Salmonella* culture-positive samples (*n* = 13 *Salmonella*-positive samples and 7 *Salmonella*-negative samples; Supplementary Tables 4, 5). When samples were looked at by date and year of collection the abundances of OTUs were again not significant after the FDR or Bonferroni corrections for multiple comparisons despite being significant at α = 0.05 (Supplementary Tables 6–9).

**Figure 6:**
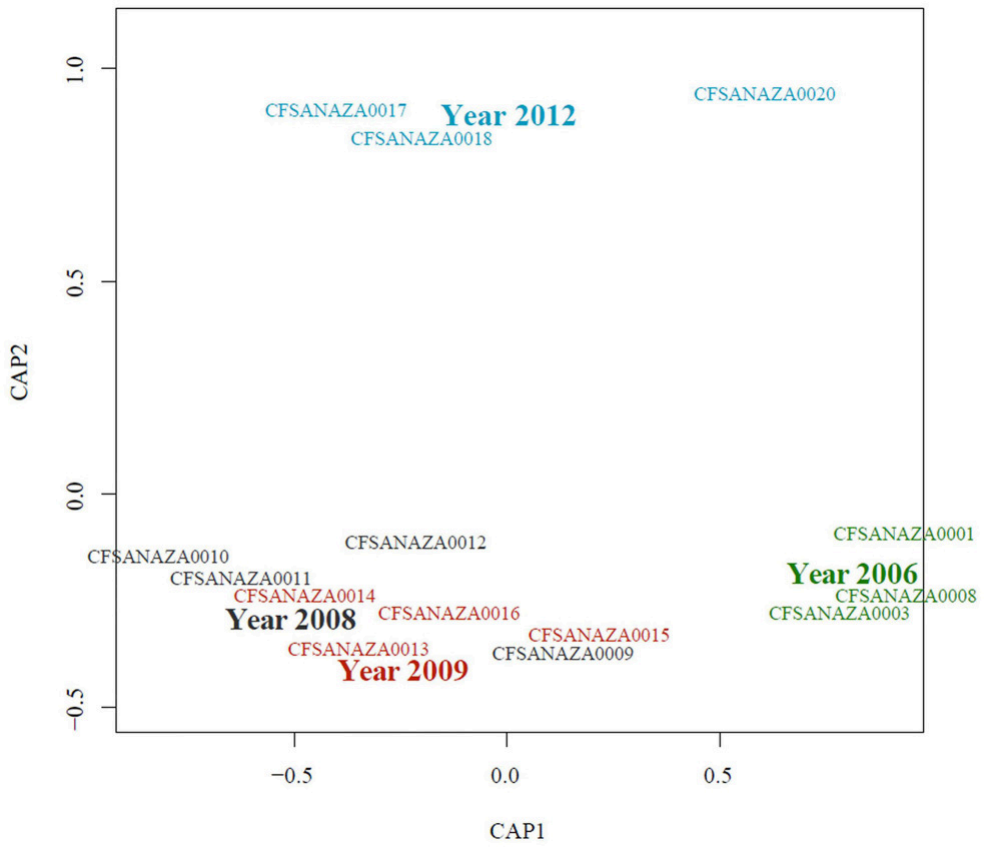
**Constrained analysis of principal coordinates (db-RDA) showing sample relationship by isolation year**. Large labels indicating the sampling year are positioned at the centroid for that level. Minor adjustments to the positions of individual sample labels were made to increase readability.

When a statistical test of significance was applied to the eigenvalues of the unifrac distance matrix using a distance-based redundancy analysis (db-RDA), we did not find evidence for a difference in microbial communities among *Salmonella* culture-positive and *Salmonella* culture-negative samples (Pseudo-*F* = 0.709, *P* = 0.523; Table 3). Because some of the *Salmonella* culture-negative samples were positive for the *S. enterica* 16S rRNA gene we grouped all *Salmonella* culture-positive and *S. enterica* 16S rRNA gene-positive (but culture-negative) samples together and re-ran the db-RDA. Similarly, no significant differences in diversity were observed between these groups (Pseudo-*F* = 0.629, *P* = 0.567). We further ran both of the previous analyses excluding the atypical microbiome samples and again did not observe any significant differences in diversity between *Salmonella*-positive and *Salmonella*-negative samples (Pseudo-*F* = 0.868, *P* = 0.536; and Pseudo-*F* = 0.971, *P* = 0.418, respectively). However, we did detect a significant difference among samples based on sampling date and year with the atypical communities included (Pseudo-*F* = 3.299, *P* < 0.05; and Pseudo-*F* = 4.385, *P* < 0.05) and excluded (Pseudo-*F* = 2.157, *P* < 0.05; and Pseudo-*F* = 2.799, *P* < 0.05; Table 3). These results appear to be driven primarily by the distinctiveness of the samples from 2006 and 2012; samples from 2008 and 2009, while distinct from those from 2006 and 2012 are not distinct from one another (Figure 6).

**Table 3:** **Results of the db-RDA tests for significant differences among samples in microbial diversity**.

	**Description**	**Results**
*Salmonella* status	*Salmonella* Culture Positive vs. *Salmonella* Culture	Pseudo-*F*: 0.709 (1, 18 Degrees of Freedom)
	Negative	Significance: 0.523
	All *Salmonella* Positive vs. *Salmonella* Culture	Pseudo-*F*: 0.629 (1, 18 Degrees of Freedom)
	Negative	Significance: 0.567
	All *Salmonella* Positive vs. *Salmonella* Culture	Pseudo-*F*: 0.971 (1, 12 Degrees of Freedom)
	Negative (excluding atypical community samples)	Significance: 0.418
	*Salmonella* Culture Positive vs. *Salmonella* Culture	Pseudo-*F*: 0.868 (1, 12 Degrees of Freedom)
	Negative (excluding atypical community samples)	Significance: 0.536
Date/Year	Date	Pseudo-*F*: 3.299 (4, 15 Degrees of Freedom)
		Significance: 0.01
	Year	Pseudo-*F*: 4.385 (3, 16 Degrees of Freedom)
		Significance: 0.004
	Date (excluding atypical community samples)	Pseudo-*F*: 2.157 (4, 9 Degrees of Freedom)
		Significance: 0.004
	Year (excluding atypical community samples)	Pseudo-*F*: 2.799 (3, 10 Degrees of Freedom)
		Significance: 0.002

*Significance is based on 999 permutations*.

## Discussion

The microbial community of the colon has evolved as a diverse consortium of microorganisms and each member inhabits a specific niche in this environment. The dynamics of the population can be impacted by changes in the diet or health of the animal and potentially by the introduction of non-native microbes that impact the resident flora by competing for resources or altering the characteristics of the environment. However, our analysis did not identify any differences in abundance of taxa or diversity of communities between dairy cows shedding *S*. Kentucky or *S*. Cerro at the time of sample collection and those that were not detected shedding these serotypes. These data suggest no major shifts in the community structure associated with the presence/absence of these frequent dairy cow-associated serotypes.

One model of bacterial modulation of the intestinal microbiome is that infection causes an immune response, such as inflammation, by the host (Lupp et al., 2007; Stecher et al., 2007). This in turn may alter the microbial community structure, most likely by selecting for those microorganisms that can withstand the pressures of the inflammation response or benefit directly from chemical changes produced by the host response. The two serotypes isolated from cows in this study encode virulence factors that are involved in mammalian infection, but their interactions with the bovine intestinal epithelium are not well-understood. In a cell-culture assay with human intestinal Caco-2 cells *S*. Cerro was shown to exhibit reduced invasiveness compared to other highly pathogenic serotypes such as *S*. Typhimurium and *S*. Newport (Rodriguez-Rivera et al., 2014). It is not known if an immune response is elicited from the bovine host during an *S*. Cerro and *S*. Kentucky infection; however, cows typically do not demonstrate observable symptoms of an infection when shedding these two serotypes suggesting that no immune response is elicited. In contrast, *S*. Typhimurium, a known pathogen of cows, causes observable signs of an infection and induces a host inflammation response in mammals, a process that has been shown to alter the resident microbiota of the mammalian host (Stecher et al., 2007).

Other models of microbial interaction in the gut include indirect interaction through resource competition, direct interaction between cells via secretion of antimicrobial compounds by one cell to interfere with the life cycle of another cell, or resource partitioning (Fredrickson and Stephanopoulos, 1981; Hibbing et al., 2010; Reeves et al., 2011; Russell et al., 2011). Based on the abundance profiles of fecal samples in this study, the hindgut is dominated by Firmicutes, and Bacteroidetes. This profile is typical of the mammalian intestinal tract (Ley et al., 2008). It is unlikely that the introduction of *S*. Kentucky or *S*. Cerro into this environment would result in a dramatic change in the abundance of the established core phyla. However, we also did not observe a consistent change in the less frequently detected taxa among all *Salmonella*-positive cows indicating that these salmonellae are not outcompeting or displacing other resident organisms in the gut. Interestingly, a previous study of significantly less sequencing depth and conducted on a *Salmonella*-endemic cattle herd similarly was unable to detect taxa that either prevented or resulted in *Salmonella* colonization of the bovine gut (Patton et al., 2009). The results of our study confirm this.

The lack of statistically noticeable differences in the microbial communities in the hindgut of cows shedding these *S*. Cerro or *S*. Kentucky supports the postulation that these serotypes can be commensal members of the bovine hindgut community. This is further evidenced by both the frequent occurrence of these serotypes among dairy herds and the absence of clinical symptoms in infected cows (Huston et al., 2002; Fossler et al., 2004; Van Kessel et al., 2012). There is, however, anecdotal evidence of some strains of *S*. Kentucky infections resulting in mild infections (data not shown) and *S*. Cerro has been recovered from cows with clinical signs of gastrointestinal distress (Cummings et al., 2010). Further, all *S*. Kentucky strains isolated from this study farm were ST152 isolates, while the distantly related *S*. Kentucky ST198 isolates are more frequently isolated from human clinical cases and encode different putative virulence factors which may result in different ecological interactions/associations in the bovine hindgut (Haley et al., unpublished data). These previously identified symptomatic infections caused by *S*. Cerro and *S*. Kentucky may be due to the expression of non-conserved virulence mechanisms found in a small subset of the global population of these serotypes, co-infections with one of many other bovine pathogens, or individual host susceptibility to infections by these serotypes. This needs to be further investigated.

Interestingly, temporal dynamics may be a significant determinant of diversity given that within this study microbial communities differed among samples based on sampling dates/years. Potential explanations for the importance of sampling time include changes in diet, the surrounding environment, or other not-yet-determined drivers of microbial community change. Several of these factors, specifically management factors would potentially impact all members of the herd and therefore their influence may result in similar hindgut community shifts. Diet, for example, has been observed to influence the community structure of the gut (Callaway et al., 2009; Wu et al., 2011; Wells et al., 2013; Kim et al., 2014) and changes in feed source and diet composition may result in structural shifts in the gut community across the herd. However, this relationship has not been observed in all bovine fecal community structure studies (Rudi et al., 2012). Although at each time point the gut communities of different cows were determined, other studies have shown similar changes over time in animals and non-animal environments (Jones et al., 2010; Caporaso et al., 2011; Rudi et al., 2012; Ottesen et al., 2015). This, coupled with the observed change in the core taxa by sampling date, indicates the dairy cow hindgut community is somewhat dynamic while retaining a smaller core set of taxa that remain relatively stable over time. Further work will need to be conducted to determine what influences this fluidity and the introduction or extinction of taxa within a herd.

Another aim of this study was to describe the composition of the herd hindgut community as well as the core community of this environment at several taxonomic levels. The microbial community profiles of the cows in this study were consistent with those of other mammals in which high levels of Firmicutes and Bacteroidetes, and lower levels of Proteobacteria for most samples, were observed indicating a general trend at higher taxonomic levels that may be conserved among mammals (Ley et al., 2008). Results of this study are, for the most part, consistent with those of others studies of the bovine gastrointestinal tract (Dowd et al., 2008; Patton et al., 2009; Callaway et al., 2010; Durso et al., 2010; Shanks et al., 2011; Rice et al., 2012; Kim et al., 2014; Mao et al., 2015; Myer et al., 2015; Kim and Wells, 2016). Although general trends were observed, variations in the abundance of the most frequently detected taxa were observed among animals. For example, there was a wide variation in the Firmicutes:Bacteroidetes ratio, a common metric used in gut health analyses and the core communities found in the hindgut changed slightly over time. For the most part, changes in the abundance of taxa could not be attributed to any group/treatment (such as date, year, or *Salmonella* status), indicating that differences in taxon abundance occurred within the individual, not the group. Further, six cows were observed to have high levels of Proteobacteria (specifically *Psychrobacter* spp.) compared to other taxa. It is not clear why some samples had particularly high counts of *Psychrobacter* spp. compared to others on the farm and compared to similar studies investigating the microbial communities of beef cattle. These organisms have been detected in the non-culturable portion of the rumen microbiome (Creevey et al., 2014) as well as the nasopharyngeal environment of feedlot cattle (Holman et al., 2015), cow manure compost (Zhao et al., 2013), and raw milk (Delbès et al., 2007; Quigley et al., 2013), so they appear to be ubiquitous in the dairy farm environment. However, it is not known whether the high levels of *Psychrobacter* spp. in a few individuals in this study were due to overgrowth in the more aerobic sections of the gut, or sampling bias, sample contamination from another body part of the cow or the surrounding environment. All cows were sampled following the same protocols, by the same investigators with sterile gloves, and for the purposes of this study only the hindgut was sampled via the rectum. A previous study by Brulc et al. (2009) described the rumen microbiome of a single cow, out of a study of three cows, in which *ca*. 75% of the 16S rRNA gene reads were *Psychrobacter* spp. We are not able to draw any conclusions as to why these individual cows had high Proteobacteria concentrations. Further work would need to be conducted to determine how often the colonic microbial community of cows has high levels of *Psychrobacter* spp. and the impact that management practices, such as feed, or presence of other genetic components have on this and other organisms.

Although detected in most samples, *Enterobacteriaceae* were a relatively small family within the microbial community. This is likely an issue of sequencing depth and/or potential PCR bias rather than true presence/absence. *Salmonella* was rarely detected even in cows from which *Salmonella* isolates were obtained. The isolates were obtained through selective enrichment and, although not guaranteed, *Salmonella* concentrations in the feces were low. It is possible that those serotypes that cause symptomatic infections in cows cause shifts within the gut population and have an increased concentration of detectable *Salmonella*-cells compared to those of asymptomatic cows. Further studies should be conducted to further evaluate this in dairy cows.

Overall, results show similarities in the community taxa profiles of dairy and beef cattle with some differences possibly due to differing management practices and diet that are varied over time. Other studies have demonstrated that cohabitating cows have noticeably different hindgut community structures (Durso et al., 2010) indicating that individual animal attributes may greatly influence this community. Results of our work demonstrate the same trend for dairy cows within the same herd. The absence of significant shifts in the hindgut communities in the presence of *S*. Cerro or *S*. Kentucky provides further evidence of these serotypes, which are frequently isolated from asymptomatic cows in the United States, as being commensal members of the bovine hindgut with little observable impact on the microbial population within the community.

## Author contributions

BH and SG conducted laboratory analysis. JP and BH conducted data analysis. BH, AO, JK, and JV designed the analysis. BH, JP, SG, AO, JK, and JV wrote the article.

### Conflict of interest statement

The authors declare that the research was conducted in the absence of any commercial or financial relationships that could be construed as a potential conflict of interest. The reviewer AK and handling Editor declared their shared affiliation, and the handling Editor states that the process nevertheless met the standards of a fair and objective review.

## Supplementary material

Supplementary Table 1 **Composition of the core microbiome at various taxonomic levels**.

Supplementary Table 2 **Results of Kruskal–Wallis test of differences in OTU abundances based on ***Salmonella*** status (*Salmonella* culture-positive vs. *Salmonella* culture-negative)**.

Supplementary Table 3 **Results of Kruskal–Wallis test of differences in OTU abundances based on ***Salmonella*** status (*Salmonella* culture-positive vs. *Salmonella* culture-negative) excluding “atypical community” samples**.

Supplementary Table 4 **Results of Kruskal–Wallis test of differences in OTU abundances based on ***Salmonella*** status (culture-positive and 16S rRNA-positive vs. culture-negative and 16S rRNA-negative)**.

Supplementary Table 5 **Results of Kruskal–Wallis test of differences in OTU abundances based on ***Salmonella*** status (culture-positive and 16S rRNA-positive vs. culture-negative and 16S rRNA-negative) excluding “atypical community” samples**.

Supplementary Table 6 **Results of Kruskal–Wallis test of differences in OTU abundances by year**.

Supplementary Table 7 **Results of Kruskal–Wallis test of differences in OTU abundances by year excluding “atypical community” samples**.

Supplementary Table 8 **Results of Kruskal–Wallis test of differences in OTU abundances by date**.

Supplementary Table 9 **Results of Kruskal–Wallis test of differences in OTU abundances by date excluding “atypical community” samples**.
